# Primary care physicians' reported use of pre-screening discussions for prostate cancer screening: a cross-sectional survey

**DOI:** 10.1186/1471-2296-10-19

**Published:** 2009-03-18

**Authors:** Suzanne K Linder, Sarah T Hawley, Crystale P Cooper, Lawrence E Scholl, Maria Jibaja-Weiss, Robert J Volk

**Affiliations:** 1 Department of Family and Community Medicine, Baylor College of Medicine, Houston, USA; 2 Center of Health Promotion and Prevention Research, The University of Texas School of Public Health, Houston, USA; 3 Division of General Medicine, Ann Arbor VAMC, University of Michigan, Ann Arbor, USA; 4 Soltera Center for Health Communication Research, Tucson, USA; 5 Macro International Inc, Atlanta, USA; 6 Houston Center for Education and Research on Therapeutics, Department of General Internal Medicine, The University of Texas M D Anderson Cancer Center, Houston, USA

## Abstract

**Background:**

Professional medical organizations recommend individualized patient decision making about prostate cancer screening. Little is known about primary care physicians' use of pre-screening discussions to promote informed decision making for prostate cancer screening. The aim of this study is to explore physicians' use of pre-screening discussions and reasons why physicians would or would not try to persuade patients to be screened if they initially refuse testing.

**Methods:**

Primary care physicians completed a self-administered survey about prostate cancer screening practices for informed decision making.

**Results:**

Sixty-six physicians (75.9%) completed the survey, and 63 were used in the analysis. Thirteen physicians (20.6%) reported not using prescreening discussions, 45 (71.4%) reported the use of prescreening discussions, and 3 (4.8%) reported neither ordering the PSA test nor discussing it with patients. Sixty-nine percent of physicians who reported not having discussions indicated they were more likely to screen African American patients for prostate cancer, compared to 50% of physicians who reported the use of discussions (Chi-square(1) = 1.62, p = .20). Similarly, 91% of physicians who reported not having discussions indicated they are more likely to screen patients with a family history of prostate cancer, compared to 46% of those who reported the use of discussion (Chi-square(1) = 13.27, p < .001). Beliefs about the scientific evidence and efficacy of screening, ethical concerns regarding patient autonomy, and concerns about time constraints differed between physicians who would and would not try to persuade a patient to be tested.

**Conclusion:**

Although guidelines recommend discussing the risks and benefits of prostate cancer screening, physicians report varying practice styles. Future research needs to consider the nature of discussions and the degree to which informed decision making is being achieved in clinical practice.

## Background

Prostate cancer is the most commonly diagnosed non-skin cancer and the second leading cause of cancer death among United States men [1]. The 2008 United States Preventive Services Task Force (USPSTF) evidence report found insufficient evidence to recommend for or against routine screening using the prostate-specific antigen (PSA) test or digital rectal examination (DRE) [2]. Professional medical organizations recommend informing men about the potential harms and benefits of prostate cancer screening, which is commensurate with informed decision making (IDM) for prostate cancer screening [3-5].

Research about IDM for prostate cancer screening has largely focused on decision aids and patient involvement in the decision-making process [6,7]. Little is known about physicians' practice styles regarding IDM for prostate cancer screening. Purvis Cooper et al. [8] conducted 14 telephone-based focus groups with 75 primary care physicians (PCPs) in 35 states and identified two predominant practice styles. Non-routine screeners included physicians who offered PSA testing without recommending for or against it. Most non-routine screeners reported having pre-screening discussions with patients about PSA testing. Routine screeners included physicians who recommended PSA testing, and few of them reported having pre-screening discussions.

Building on the qualitative work of Purvis Cooper et al. [8], the aim of this study is to explore the use of pre-screening discussions by PCPs to promote IDM. Secondarily, we examined the validity of a self-report indicator to classify physicians as those reporting use of pre-screening discussions (D = discussing) and those who do not (ND = non-discussing). We also explored reasons why physicians would or would not try to persuade patients to be screened if they initially refused PSA testing, and how their screening practices differ for men in high risk groups (i.e., African American race, and family history of prostate cancer).

## Methods

### Participants and procedures

In February 2004, 87 PCPs from a university-based family medicine clinic and six community health centers in Houston, Texas were invited to complete self-administered surveys. Reminder letters and surveys were sent to nonresponders after three weeks. The Baylor College of Medicine Institutional Review Board approved this study for use of human subjects in research. PCPs provided acknowledgement of consent by returning the surveys.

### Development and validation of questionnaire survey

A single-item indicator of practice style was developed based on the pre-screening discussion findings of routine and non-routine screeners from Purvis Cooper, et al. [8]. The context was screening an age-appropriate man with no other risk factors for prostate cancer using the PSA test. The practice style indicator reflected whether clinicians routinely reported use of pre-screening discussions and, among clinicians who do (D), their usual decision making role (recommend for, recommend against, or let patient decide). Face validity was established through iterative feedback from 3 PCPs.

Two questions were included to assess screening practices for high risk men (i.e., African American men and men with a family history of prostate cancer) compared to other age-appropriate men without the risk factor. Response options were "more likely to screen," "less likely to screen," or "screening practices the same." PCPs also were asked if they would try to persuade a man to have the PSA test if he initially refused, and offer reasons why or why not.

Validity of the practice style indicator was assessed by asking PCPs how often they order PSA tests for patients who were screening candidates (response options, "never" to "always" on a 6-point Likert scale).

### Statistical analysis

We calculated the proportion of D and ND respondents. Validity of the practice style indicator question was examined using Kruskal-Wallis one-way analysis of variance to compare physicians' reported frequencies of discussing screening harms and benefits and how often patients were screened with PCPs' responses to the practice style indicator. Chi-square tests were used to explore differences in screening frequency for high risk patients across practice styles. Descriptive statistics were performed with SPSS 13.0.

### Responses to open-ended questions

Responses to open-ended questions about persuading a man to have the PSA test after he initially refusal were reviewed by two authors and each response was coded into themes concerning cancer beliefs. The themes were grouped by those who would and would not try to persuade the patient to have the test.

## Results

Sixty-six (75.9%) of the 87 PCPs completed the survey. Sixty-three (72.4%) were included in the analysis, including 35 family physicians and 28 general internists. The three excluded were 2 urgent care physicians and 1 working in psychiatry.

**Figure 1:**
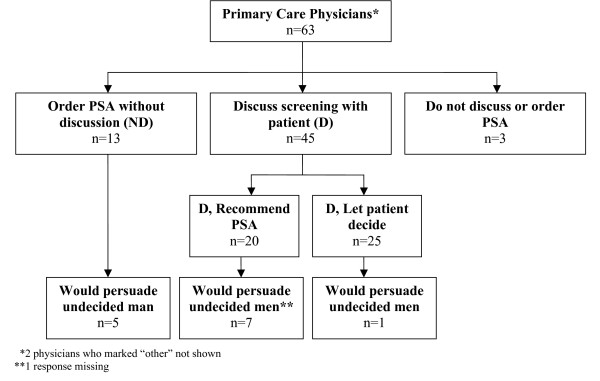
**Physicians who would persuade men to have the PSA test after initial refusal**.

Thirteen (20.6%) respondents were classified as ND providers, 45 (71.4%) were D providers, and 3 (4.8%) providers reported neither ordering PSA tests nor discussing testing with patients. Two physicians marked "other." Among the D providers, 20 (44.4%) reported they recommend screening and 25 (55.6%) responded they would let patients decide after discussing harms and benefits (see Figure 1).

**Figure 2:**
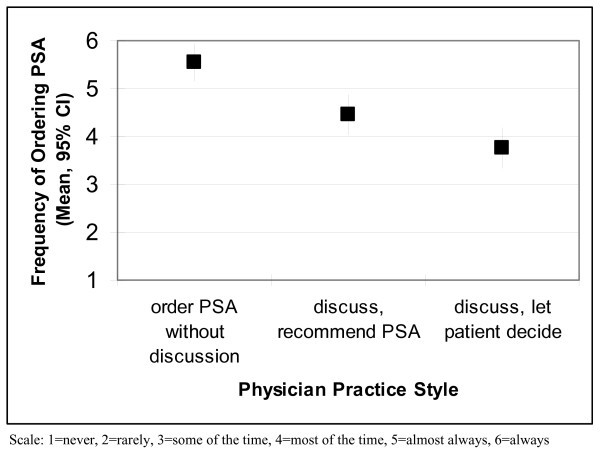
**Reported frequency of ordering PSA tests by physician practice style**.

Figure 2 shows the relationship between self-reported practice style and the frequency of ordering the PSA test. The reported frequency of ordering PSA tests was highest among ND PCPs followed by D PCPs who recommend PSA testing for their patients. D PCPs who let patients decide about testing reported the lowest frequency of ordering PSA tests.

Figure 1 shows responses to persuading a man to have a PSA test after he initially refuses by self-reported practice style. Of the 13 ND PCPs, 5 (38.5%) indicated they would persuade the man. Similarly, of the 19 D PCPs who recommend screening, 7 (36.8%) said they would try to persuade the man (one response was missing). Of the 25 D PCPs who let the patient decide, only 1 (4%) would try to persuade the man. Themes from the responses to the open-ended question about why physicians would or would not try to persuade a patient to be tested if he refused the PSA test are given in Table 1. Physicians who would try to persuade a patient to be tested believed in the efficacy of screening and PSA testing, specifically, and were concerned that prostate cancer often presents asymptomatically. These PCPs felt screening should be part of routine care. Physicians who would not try to persuade a patient to be tested questioned the lack of scientific evidence and efficacy of the PSA test. Some of these PCPs were concerned about treatment side effects and others reported there was insufficient time to discuss screening during clinical encounters. They also noted patient autonomy as an important factor.

**Table 1:** Responses to question about persuading a patient to be screened who refuses the PSA test.^±^*

**Would try to persuade patient to have the PSA test** **(n = 13)**	**Would not try to persuade patient to have the PSA test** **(n = 45)**
**Belief in Early Detection**	**Patient Autonomy**
"Because prostate cancer found early is curable."	"The patient is the ultimate decision maker."
"How else could you be diagnosed and treated?"	"The patient has the final say in his care."
	"Patient has the right to refuse after the discussion of the benefit and risk of PSA screening."
**Asymptomatic Presentation**	**Lack of Scientific Evidence**
"Because prostate cancer could be asymptomatic."	"No good evidence that screening prevents morbidity & mortality."
"I have a number of asymptomatic patients with increased PSA. Therefore, prostate cancer."	
**Belief in Efficacy of the Test**	**Questionable Efficacy of Test**
"Relatively low number false positives."	"The test is not specific enough to recommend without reservation in low risk people."
"Personally I believe in the benefit."	"Test has too many false positives."
**Part of Routine Care**	**Time Constraints**
"Usually doing other lab work – one more tube if positive then he can consider further evaluation if desired."	"Too time intensive."
	**Concerns about Side Effects**
	"The complications of biopsy and treatment might outweigh the benefit."

^± ^Data from a university-based family medicine clinic and six community health centers in Houston, TX collected in February 2004.

*One response missing.

The majority of PCPs report being more likely to screening African American men; screening behavior appeared similar across practice styles (see Table 2). In contrast, family history was particular important for PCPs who routinely discuss prostate cancer screening with their patients. Specifically, D PCPs seemed more likely to report screening their patients for prostate cancer if they have a family history, compared to ND PCPs.

**Table 2:** Reported physician screening practice for high risk men, by physician practice style.*

	Physician Practice Style			*P*-value
		
	Order PSA without discussion	Discuss, recommend PSA	Discuss, let patient decide	
Are your prostate cancer screening practices different for African American Men?				
No difference in screening practice	46.2% (6)	35.0% (7)	28.0% (7)	0.54
More likely to screen	53.8% (7)	65.5% (13)	72.0% (18)	
Are your prostate cancer screening practices different for men with a family history of prostate cancer?				
No difference in screening practice	50.0% (7)	20.0% (4)	3.7% (1)	0.01
More likely to screen	50.0% (7)	80.0% (16)	96.3% (26)	

*P*-values from Chi-square test. Sample size for each cell given in parentheses.

* Data from a university-based family medicine clinic and six community health centers in Houston, Texas collected in February 2004.

## Discussion

### Summary of findings

We found support for two general physician practice styles related to prostate cancer screening informed decision making. ND providers who reported not using pre-screening discussions appear to generally order PSA tests without discussing potential harms and benefits. D providers who reported using pre-screening discussions also reported to discuss harms and benefits of testing, although their decision-making roles varied; specifically, some let patients decide while others recommend testing. None of the ND or D physicians reported recommending against the test. In addition, the practice style question provided evidence for validity when compared to physicians' reported frequencies of ordering PSA tests.

It is surprising that most physicians in this study reported discussing screening harms and benefits given the substantial barriers to promoting IDM in clinical practice [9]. It also is interesting that many of the D providers still recommend screening to their patients. This finding may reflect some of the tension between professional guidelines and concerns about malpractice resulting from missed cancer diagnoses [10].

Risk factors appear to play a role in how likely D physicians are to screen patients for prostate cancer. The majority of physicians appear more likely to screen African American men than other men. Similarly, over 80% of physicians whose practice pattern for patients with no risk factors is to discuss prostate cancer screening reported being more likely to screen patients with family histories compared with other patients.

When faced with a patient who initially declines PSA testing, some important beliefs help explain physicians' responses. Beliefs about scientific evidence and efficacy of screening, ethical concerns regarding patient autonomy, and pragmatic concerns (e.g., time constraints) seem to be different between physicians who would and would not try persuading patients to be tested.

#### Limitations

Limitations of this study include using self-report data instead of observing discussions between physicians and patients. Some physicians reported discussing screening harms and benefits with patients, but the content of such communications was not investigated. Although only three PCPs reviewed the practice style question for content validity, evidence for validity was also seen when compared to a frequency of PSA testing question. The results of this study are limited a sample of university-based physicians and may not be generalizable to PCPs in non-academic settings.

#### Current Literature about Physicians' IDM Practice Style for Prostate Cancer Screening

Existing research has been very limited about physicians' practice styles regarding IDM for prostate cancer screening. In comparison to the qualitative work of Purvis Cooper et al [6], our study also found predominate practice styles for prostate cancer screening in regards to routine and non-routine screening, but also practice styles appear to differ by those who have pre-screening discussions and those who do not. Additionally, many of the qualitative themes about beliefs about prostate cancer screening that emerged from Purvis Cooper's study were also found in the open responses to persuading men about the PSA test in our study.

## Conclusion

Although guidelines recommend discussing potential harms and benefits of prostate cancer screening, physicians report varying prostate cancer screening practice styles. Future research needs to consider the nature of patient-provider discussions and the degree to which IDM is achieved in clinical practice. Additionally, IDM practice styles for prostate cancer screening should be explored to see if styles differ by provider characteristics such as age, gender, practice setting, and beliefs about prostate cancer screening.

## Competing interests

The authors declare that they have no competing interests.

## Authors' contributions

SKL has made a contribution to conception and design, carried out acquisition of data, analysis, and interpretation, and drafting the manuscript. STH, MJW, CPC, LES, and RJV have made a contribution to the conception and design, and were involved in revisions and drafting of the manuscript. All authors read and approved the final manuscript.

## Pre-publication history

The pre-publication history for this paper can be accessed here:


